# Identifying cirrhosis, decompensated cirrhosis and hepatocellular carcinoma in health administrative data: A validation study

**DOI:** 10.1371/journal.pone.0201120

**Published:** 2018-08-22

**Authors:** Lauren Lapointe-Shaw, Firass Georgie, David Carlone, Orlando Cerocchi, Hannah Chung, Yvonne Dewit, Jordan J. Feld, Laura Holder, Jeffrey C. Kwong, Beate Sander, Jennifer A. Flemming

**Affiliations:** 1 Department of Medicine, University of Toronto, Toronto, Canada; 2 Institute of Health Policy, Management and Evaluation, University of Toronto, Toronto, Canada; 3 Institute for Clinical Evaluative Sciences, Toronto, Canada; 4 McGill University Medical School, Montreal, Canada; 5 Queen’s University School of Medicine, Kingston, Canada; 6 University Health Network, Toronto, Canada; 7 Institute for Clinical Evaluative Sciences, Kingston, Canada; 8 Public Health Ontario, Toronto, Canada; 9 Dalla Lana School of Public Health, Toronto, Canada; 10 Department of Family and Community Medicine, University of Toronto, Toronto, Canada; 11 Toronto Health Economics and Technology Assessment Collaborative, Toronto, Canada; 12 Department of Medicine and Department of Public Health Sciences, Queen`s University, Kingston, Canada; Nihon University School of Medicine, JAPAN

## Abstract

**Background:**

To evaluate screening and treatment strategies, large-scale real-world data on liver disease-related outcomes are needed. We sought to validate health administrative data for identification of cirrhosis, decompensated cirrhosis and hepatocellular carcinoma among patients with known liver disease.

**Methods:**

Primary patient data were abstracted from patients of the Toronto Center for Liver Disease with viral hepatitis (2006–2014), and all patients with liver disease from the Kingston Health Sciences Centre Hepatology Clinic (2013). We linked clinical information to health administrative data and tested a range of coding algorithms against the clinical reference standard.

**Results:**

A total of 6,714 patients had primary chart data abstracted. A single physician visit code for cirrhosis was sensitive (98–99%), and a single hospital diagnostic code for cirrhosis was specific (91–96%). The most sensitive algorithm for decompensated cirrhosis was one cirrhosis code with any of: a hospital diagnostic code, death code, or procedure code for decompensation (range 88–99% across groups). The most specific was one cirrhosis code and one hospital diagnostic code (range 89–98% across groups). Two physician visit codes or a single hospital diagnostic code, death code, or procedure code combined with a code for cirrhosis were sensitive and specific for hepatocellular carcinoma (sensitivity 94–96%, specificity 93–98%).

**Conclusion:**

These sensitive and specific algorithms can be used to define patient cohorts or detect clinical outcomes using health administrative data. Our results will facilitate research into the adequacy of screening and treatment for patients with chronic viral hepatitis or other liver diseases.

## Introduction

In 2016, 1.26 million people worldwide died of cirrhosis and chronic liver diseases, and their complications.[1] Hepatocellular carcinoma (HCC) mortality rates are rising faster than those from any other malignancy.[2] Globally, viral hepatitis secondary to hepatitis B and C virus infection underlies 55% of cirrhosis-related deaths and 61% of deaths from HCC.[1] Yet, many patients with liver disease remain undiagnosed, largely because they remain asymptomatic until a late stage.[3, 4] Of late, much progress has been made in the prevention and treatment of viral hepatitis. Many jurisdictions have advanced the timing of immunization against hepatitis B virus (HBV) from early adolescence to infancy.[5, 6] Further, new treatments for chronic hepatitis C virus (HCV) infection have enabled large numbers of patients to achieve sustained virologic response, a marker of long-term clinical cure.[7–10] Finally, our understanding of the epidemic of non-alcoholic fatty liver disease (NAFLD) in North America is just beginning and the natural history of this disease is still not completely defined.

To evaluate the epidemiology and the long-term clinical effectiveness of treatments and screening programs for chronic liver diseases, it is essential to accurately detect clinical outcomes such as cirrhosis, decompensated cirrhosis, and HCC. Although a registry of patients could be used to measure long-term treatment effects, it is ill-suited to study the overall burden or healthcare utilization related to liver disease since liver-related outcomes can take decades to occur. Longitudinal, systematically collected information from large cohorts of patients with chronic liver disease is needed.

Routinely collected health administrative data enable efficient research on real-world outcomes of patients with liver disease, while offering objective evidence of past healthcare utilization. Although several studies have validated data algorithms for identifying cirrhosis, decompensated cirrhosis, and HCC, these have been limited to International Classification of Diseases 9^th^ revision (ICD-9) codes in the healthcare system of the United States.[11–17] In Canada, all hospitalization data have been coded using the ICD-10 system since 2002 and as of as of October 2015, all hospital discharge information in the United States have also been coded using the ICD-10 system.[18, 19] While administrative data codes have been used in outcomes research[20, 21], there are no existing validation studies for liver-related outcomes in Canadian patients and no validation studies using ICD-10 codes in the United States.

The primary objective of this study was to measure the validity of combined ICD-9 and ICD-10 health administrative data codes for detecting cirrhosis, decompensated cirrhosis, and HCC in patients with known chronic liver disease. This will facilitate the study of the long-term effects of antiviral treatment or policy changes relating to this patient population. Furthermore, as the epidemiology of chronic liver diseases such as NAFLD and alcohol-related disease have a different natural history than viral hepatitis, we also aimed to assess their broader validity in a group of patients with liver diseases of all causes.

## Methods

### Setting

The validation cohorts consisted of patients from two different university-affiliated tertiary care hepatology clinics in Ontario, Canada: The Toronto Centre for Liver Disease (TCLD) at the University Health Network (UHN) located in Toronto and the Liver Disease Clinic at the Kingston Health Sciences Centre (KHSC) located in Kingston. Both clinics are staffed by subspecialty trained academic hepatologists and receive patient referrals for patients with acute or chronic liver diseases. At both sites, clinicians employ a standardized computerized form for clinical data entry. Patient status, including test results and treatments, are updated at every encounter. UHN and KHSC clinical records include information on patient demographics, most responsible diagnosis, laboratory data, imaging data, endoscopic reports, pathology data, non-invasive fibrosis assessment tests and results, and any hepatic decompensation events.

### Administrative databases

The Institute for Clinical Evaluative Sciences holds health administrative data for all Ontario residents with provincial health insurance. Data on demographics, physician visits, emergency department visits, hospital admissions and procedures are linked using an encrypted patient identifier.[22]

The Ontario Health Insurance Program (OHIP) database contains all billing claims made by physicians.[23] The Canadian Institute for Health Information’s Discharge Abstract Database contains information for all admissions to acute care hospitals in Ontario[24], and the National Ambulatory Care Reporting System database contains information on emergency department and day surgery visits.[22] The Office of the Registrar General Death Database contains the cause of death for all deaths in the province.[25] The Ontario Cancer Registry includes detailed clinical information on malignancies such as anatomical site and tumour histology.[26]

### Study population

We identified patients for inclusion in the chronic HBV and HCV cohorts using a two-step process. First, any patient followed at the TCLD with an HBV or HCV treatment status, positive HBV or HCV serology, positive HBV DNA, or positive HCV RNA, with a clinic visit between April 2006 and March 2014 were selected for further review. We then reviewed the charts to confirm chronic HBV or HCV status rather than resolved prior infection (patients with the latter were not included in the study cohorts). Patients who had evidence of ongoing infection with HBV or HCV at any time from their first clinic visit to March 31^st^ 2014 were included in the study. The cohort of patients from KHSC comprised consecutive patients seen at the KHSC Liver Clinic from May through August 2013. Patients in the KHSC cohort had liver disease of viral and non-viral etiology. Across all groups, any patient that could not be linked to administrative data holdings was excluded from the study.

### Reference standard: Clinical outcomes

Outcomes evaluated in this study included cirrhosis, decompensated cirrhosis, and hepatocellular carcinoma. Two trained medical graduates (FG at TCLD and DC at KHSC) reviewed the charts to identify clinical outcomes.

Decompensated cirrhosis was identified based on the presence of any of the following in the clinical record: ascites, bleeding varices, encephalopathy, use of spironolactone without alternative indication, or explicit mention of decompensated cirrhosis. Cirrhosis was identified based on any of the decompensated cirrhosis criteria, or explicit mention of cirrhosis, non-bleeding varices, or use of nadolol without alternative indication. In addition, cirrhotic appearance on ultrasound, a liver biopsy result of F4 fibrosis, or a non-invasive test result consistent with F4 fibrosis were also considered diagnostic of cirrhosis. Hepatocellular carcinoma was identified based on explicit mention anywhere in the clinical note. Uncertain cases were reviewed and classified by a hepatologist (JJF at UHN or JAF at KHSC).

A 5% random sample of charts was re-abstracted by a general internist at TCLD (LLS) and a hepatologist at KHSC (JAF). Agreement beyond chance on the outcome ascertainment by both abstracters was measured using Cohen’s kappa.

### Administrative data outcomes

The primary outcomes of cirrhosis, decompensated cirrhosis and HCC were defined using relevant physician visit, emergency department visit, hospital diagnosis, procedure, death and pathology codes (Table 1). A secondary outcome of 2-year all-cause mortality following last clinic visit was reported as an overall measure of patient severity of illness.

**Table 1:** Administrative data codes used to identify cirrhosis, decompensated cirrhosis and hepatocellular carcinoma. OHIP = Ontario Health Insurance Plan, ICD-9 = International Classification of Diseases, 9^th^ Revision, ICD-10 = = International Classification of Diseases, 10^th^ Revision, CCP = Canadian Classification of Diagnostic, Therapeutic, and Surgical Procedures, CCI = Canadian Classification of Health Interventions.

**Cirrhosis**	
Physician Visit Code	OHIP: 571
Hospital Diagnostic Codes	ICD-9 : 456.1, 571.2, 571.5 ICD-10: I85.9, I98.2, K70.3,K71.7, K74.6
**Chronic Liver Disease**	
Hospital Diagnostic Codes	ICD-9: 070.2X, 070.3X, 070.4X, 070.5X, 070.6, 070.9, 571.0, 571.3, 571.4X, 571.8, 573.1, 573.3 ICD-10: K70.0, K70.2, K73.X, K754, K758, K75.9, K76.0, B18.0, B18.1, B18.2, B18.8, B18.9
**Complications of Cirrhosis**	
Diagnostic and Procedure Codes	ICD-9: 155.0, 572.2, 572.3, 572.4, 456.0, 456.2, 782.4, 789.5, V427 ICD-10 : C22.0,C22.9, 81703,81803, I85.0, I86.4, I98.20, I98.3, K721, K729, K76.6, K76.7, R17, R18, T86.400, T86.401, Z76804, Z944 CCP: 1006, 6691,62.40, 62.41, 62.49 CCI: 1.NA.13.BA-FA, 1.NA.13.BA-X7, 1.NA.13.BA-BD, 1.KQ.76GP-NR, 1.OT.52.HA,1.OA.59^^, 1.OA.85^^ OHIP: 155, J057, J069, Z591, S294, S295, S265, S266
**Decompensated Cirrhosis**	
Hospital Diagnostic Codes	ICD-9: 456.0, 456.2, 572.2, 572.3, 572.4, 782.4, 789.5 ICD-10 : I85.0, I86.4, I98.20, I98.3, K721, K729, K76.6, K76.7, R17, R18
Procedure Codes	CCI: 1.NA.13.BA-FA, 1.NA.13.BA-X7, 1.NA.13.BA-BD, 1.KQ.76GP-NR, 1.OT.52.HA CCP: 1006, 6691 OHIP: J057, Z591
Death Codes	ICD-9: 5715, 5712, 5722, 5723, 5724, 5728, 4560 ICD-10: K721, K729, K703, K704, K717, K74, K746, K766, K767, I85X, I864, I982X, I983
**Hepatocellular Carcinoma**	
Physician Visit Code	155
Hospital Diagnostic Codes	ICD-9 : 155.0 ICD-10 : C22.0, C22.9, 81703, 81803
Procedure Codes	OHIP: J069 CCI: 1.OA.59^^
Death Codes	ICD-9: 1550 ICD-10: 81703, 81803
Ontario Cancer Registry Codes	Morphology: 81703, 81723, 81733, 81743, 81753, 81803 Topography: C220

We built a series of diagnostic algorithms for each outcome ranging from simple (e.g. a single physician visit code) to more complex. This was done to identify the most parsimonious algorithms that were also highly sensitive and/or specific. We aimed to utilize all available health administrative data, and include both hospital-based and outpatient physician visits in our algorithms. Data sources were searched for relevant codes ten years prior to two years following the date of last clinical assessment (up to March 31^st^, 2014 for TCLD and August 31^st^, 2013 for KHSC).

Cirrhosis algorithms ranged from cirrhosis codes only to combinations with codes for chronic liver disease or any complication (decompensation events, HCC or liver transplant). Algorithms were combined in such a way as to make them more sensitive (“or” combinations) or specific (“and” combinations). As physician visit codes were noted to be less specific, we aimed to increase specificity by combining two or more such codes with hospitalization codes.

Decompensation algorithms included hospital diagnostic and death codes for portal hypertension, hepatorenal syndrome, jaundice, hepatic coma, hepatic failure, bleeding esophageal varices, gastric varices (bleeding not specified), and ascites. There were no physician visit codes available for decompensation events. We included procedure codes for endoscopy or insertion of Sengstaken tube for upper gastrointestinal bleeding, transjugular intrahepatic portosystemic shunt, and paracentesis. Since several of these procedures could occur for reasons other than decompensated cirrhosis (such as bleeding from an ulcer, or ascites secondary to an extra-hepatic malignancy), we tested combinations of procedure codes with a cirrhosis code from a physician visit.

Hepatocellular carcinoma algorithms ranged from simple (a single physician visit code) to more complex. We tested several combinations in order to optimise both sensitivity and specificity. We combined physician visit codes, hospital diagnostic codes, and cause of death codes. Further, we included procedure codes for radiofrequency ablation. Since this procedure can also be used to ablate tumours outside the liver (e.g., renal tumours), we combined ablation codes with an outpatient code for cirrhosis. Finally, we tested our results with and without anatomical and pathology codes from the Ontario Cancer Registry.

### Analysis

Characteristics of patients in each validation cohort (HBV and HCV patients from TCLD, patients from KHSC) and 2-year mortality were described using univariate statistics.

We tested the performance of administrative data algorithms for cirrhosis, decompensated cirrhosis, and HCC against the clinical reference standard. Each algorithm was evaluated for sensitivity, specificity, and overall accuracy. Performance measures were reported with their 95% confidence intervals. We did not report positive and negative predictive values as these parameters are highly dependent on prevalence in the reference population, making them poorly generalizable.

Measurement of algorithm performance was performed using SAS software, version 9.4 (SAS Institute Inc., Carey, NC). This project was approved by the Research Ethics Boards of UHN and KHSC.

## Results

From April 2006 to March 2014, there were 3,502 patients with chronic HBV and 2,956 patients with chronic HCV seen at TCLD, of which 3,381 (97%) with HBV and 2,891 (98%) with HCV could be linked to administrative data. From May to August 2013, there were 444 patients seen at the KHSC Liver Clinic, of which 442 (99.5%) could be linked to administrative data. The most common causes of liver disease in KHSC patients were: HCV in 199 (45%), NAFLD in 49 (11%), alcohol-related liver disease in 37 (8%), autoimmune liver disease in 36 (8%) and HBV infection in 34 (8%).

The characteristics of TCLD patients with HBV or HCV and all KHSC patients are presented in Table 2. The patients in the validation cohorts were, on average, middle-aged (median age 48–57 years), more likely to be male (57–60%), frequently low-income (26–28%), and mostly urban (74–99%). Many TCLD patients were either still consuming alcohol or had done so regularly in the past (37% of HBV patients, 64% of HCV patients). Few patients had been hospitalized in the previous year (1–16%), however many KHSC patients had visited the emergency department (44%). Most patients had not undergone a liver biopsy, but had been assessed clinically, including using non-invasive fibrosis testing (44–75%).

**Table 2:** Characteristics of patients in the three validation cohorts, at the time of last clinical follow-up. HBV = Hepatitis B Virus infection, HCV = Hepatitis C virus infection, TCLD = Toronto Centre for Liver Disease, KHSC = Kingston Health Sciences Centre, NA = not available.

	HBV Patients, TCLD (n = 3,381)	HCV Patients, TCLD (n = 2,891)	KHSC Patients (n = 442)
**Age in years, median (IQR)**	48 (37–57)	55 (47–61)	57 (49–62)
**Sex Female,** n (%)	1,447 (43)	1,154 (40)	180 (41)
**Income quintile, n(%)** **1- Lowest** **2** **3** **4** **5- Highest**	916 (27) 767 (23) 625 (19) 573 (17) 476 (14)	742 (26) 557 (19) 513 (18) 539 (19) 517 (18)	123 (28) 90 (20) 82 (19) 79 (18) 62 (14)
**Rural, n (%)**	19 (1)	138 (5)	115 (26)
**Alcohol consumption, n (%)** **Currently drinking** **Never Used** **Stopped drinking**	847 (25) 2,115 (63) 402 (12)	853 (30) 1,004 (35) 993 (34)	N/A
**Urgent hospitalization in year prior, n(%)**	43 (1)	108 (4)	71 (16)
**ER visit in year prior, n(%)**	248 (7)	502 (17)	194 (44)
**Fibrosis Assessment, n (%)** **Non-invasive score or clinical** **Liver biopsy**	2,372 (70) 1,009 (30)	1,261 (44) 1,630 (57)	328 (74) 114 (26)
**Fibrosis Stage, n (%)** **F0** **F1** **F2** **F3** **F4**			62 (14) 43 (10) 68 (15) 36 (8) 233 (53)

Of TCLD patients with HBV infection, 669 (19%) had cirrhosis, 99 (3%) had decompensated cirrhosis and 133 (4%) had hepatocellular carcinoma at any time during follow-up. Of the patients with HCV, 1,175 (40%), 335 (11%) and 167 (6%) had a clinical diagnosis of cirrhosis, decompensated cirrhosis, and HCC, respectively. For KHSC patients, this was 233 (53%), 93 (21%) and 25 (6%) for cirrhosis, decompensated cirrhosis, and HCC, respectively. By two years following their last clinic visit, 4% (n = 140) of TCLD patients with HBV, 8% (n = 243) of TCLD patients with HCV and 12% (n = 53) of KHSC patients had died.

At re-abstraction of charts belonging to HBV patients (n = 176) from TCLD, Cohen’s Kappa was 0.94 for cirrhosis, 1 for decompensated cirrhosis and 1 for hepatocellular carcinoma. For HCV patients (n = 148), Cohen’s kappa was 0.99 for cirrhosis, 0.92 for decompensated cirrhosis, and 1 for hepatocellular carcinoma. For KHSC patients (n = 20), there was complete agreement (kappa = 1) for the presence of cirrhosis, decompensated cirrhosis and hepatocellular carcinoma.

### Algorithms for cirrhosis

A single physician visit code for cirrhosis was highly sensitive (98% for HBV, 99% for HCV and KHSC group, Table 3), while a single hospital diagnostic code for cirrhosis was specific (96% for HBV, 91% for HCV, 92% for KHSC group). Greatest specificity was achieved using a combination of a chronic liver disease code, a complication code and either 2+ physician visit codes or a single hospital diagnostic code (algorithm 13: 98% in HBV, 95% in HCV, 97% for KHSC cohort). A single hospital diagnosis code had the greatest overall accuracy in all three groups (HBV 88%, 95% CI 87–90%, HCV 84%, 95% CI 83–85%, KHSC 87%, 95% CI 84–90%).

**Table 3:** Administrative data algorithms used to identify patients with cirrhosis. Number of patients in each group with cirrhosis (reference outcome) indicated (n) at top of column. HBV = Hepatitis B Virus infection, HCV = Hepatitis C virus infection, TCLD = Toronto Centre for Liver Disease, KHSC = Kingston Health Sciences Centre, CLD = Chronic Liver Disease, Sens = sensitivity, Spec = specificity, CI = confidence interval. 1+ = code occurs on at least one date; 2+ = code occurs on at least two separate dates.

Algorithm		HBV Patients, TCLD (n = 669)	HCV Patients, TCLD (n = 1,175)	KHSC Patients (n = 233)
		Sens (95%CI), % Spec (95%CI), %	Sens (95%CI), % Spec (95%CI), %	Sens (95%CI), % Spec (95%CI), %
1	1+ Hospital Diagnosis CIRRHOSIS	57 (53–60) 96 (96–97)	73 (71–76) 91 (90–93)	77 (71–82) 92 (89–96)
2	1+ Hospital Diagnosis CIRRHOSIS or 1+ COMPLICATION	67 (63–70) 90 (89–92)	80 (78–83) 77 (75–79)	82 (78–87) 85 (80–90)
3	1+ Physician Visit CIRRHOSIS	98 (96–99) 78 (76–80)	99 (98–99) 64 (62–66)	99 (97–100) 66 (59–72)
4	1+ Physician Visit or 1+Hospital Diagnosis CIRRHOSIS	98 (97–99) 77 (75–78)	99 (99–100) 61 (59–64)	99 (97–100) 63 (57–70)
5	1+ Physician Visit or 1+ Hospital Diagnosis CIRRHOSIS and 1+ CLD	73 (70–77) 88 (87–89)	82 (80–84) 72 (70–74)	58 (52–64) 86 (81–90)
6	1+ Physician Visit or 1+ Hospital Diagnosis CIRRHOSIS or 1+ COMPLICATION	98 (97–99) 74 (72–0.75)	99 (99–100) 54 (52–57)	99 (97–100) 63 (57–70)
7	1+ Physician Visit or 1+ Hospital Diagnosis CIRRHOSIS and 1+ COMPLICATION	46 (43–50) 97 (96–97)	61 (58–63) 91 (89–92)	72 (66–78) 91 (87–95)
8	1+ Physician Visit or 1+ Hospital Diagnosis CIRRHOSIS and 1+ COMPLICATION and 1+ CLD	40 (37–44) 98 (97–98)	55 (52–58) 93 (92–94)	45 (38–51) 96 (93–98)
9	2+ Physician Visit or 1+ Hospital Diagnosis CIRRHOSIS	94 (92–95) 85 (83–86)	98 (97–99) 71 (69–73)	95 (92–98) 78 (72–84)
10	2+ Physician Visit or 1+ Hospital Diagnosis CIRRHOSIS and 1+ CLD	72 (68–75) 91 (90–92)	81 (79–83) 77 (75–79)	56 (49–62) 88 (84–92)
11	2+ Physician Visit or 1+ Hospital Diagnosis CIRRHOSIS or 1+ COMPLICATION	94 (92–96) 81 (79–82)	98 (97–99) 62 (60–64)	96 (93–98) 74 (68–80)
12	2+ Physician Visit or 1+ Hospital Diagnosis CIRRHOSIS and 1+ COMPLICATION	46 (42–50) 97 (97–98)	61 (58–63) 93 (92–94)	71 (65–77) 93 (89–96)
13	2+ Physician Visit or 1+ Hospital Diagnosis CIRRHOSIS and 1+COMPLICATION and 1+ CLD	40 (36–44) 98 (98–99)	55 (52–58) 95 (93–96)	44 (38–51) 97 (95–99)

### Algorithms for decompensated cirrhosis

The algorithm with the greatest sensitivity (88% in HBV, 92% in HCV, 99% in KHSC group), was one physician visit code for cirrhosis and any of the following: one hospital diagnostic code, death code, or procedure code (Table 4). For the HBV and HCV groups, this algorithm also had the highest overall accuracy (95%, CI 94–96% in HBV group; 88%, CI 87–90% in HCV group). The most specific algorithm was one physician visit code for cirrhosis and one hospital diagnostic code for decompensation (95% in HBV, 88% in HCV, 79% in the KHSC group); this was also the algorithm with the greatest overall accuracy in the KHSC group (83%, 95% CI 80–87%). After excluding a single hospital diagnostic code which accounted for many false positives (ICD-10 code for portal hypertension K766), the specificity of this algorithm was further improved to 98% in HBV, 95% in HCV, and 89% in KHSC groups.

**Table 4:** Administrative data algorithms used to identify patients with decompensated cirrhosis. Number of patients in each group with decompensated cirrhosis (reference outcome) indicated (n) at top of column. HBV = Hepatitis B Virus infection, HCV = Hepatitis C virus infection, TCLD = Toronto Centre for Liver Disease, KHSC = Kingston Health Sciences Centre, CLD = Chronic Liver Disease, Sens = sensitivity, Spec = specificity, CI = confidence interval. 1+ = code occurs on at least one date; 2+ = code occurs on at least two separate dates.

Algorithm		HBV patients, TCLD (n = 99)	HCV patients, TCLD (n = 335)	KHSC patients (n = 93)
		Sens (95%CI), % Spec (95%CI), %	Sens (95%CI), % Spec (95%CI), %	Sens (95%CI), % Spec (95%CI), %
1	1+ Hospital Diagnosis	85 (78–92) 95 (94–96)	90 (87–93) 88 (87–89)	99 (97–100) 79 (74–83)
2	1+ Hospital Diagnosis or 1+ Procedure	86 (79–93) 94 (93–95)	91 (88–94) 87 (86–88)	99 (97–100) 78 (74–83)
3	1+ Hospital Diagnosis or 1+ Death Code or 1+ Procedure	88 (81–94) 94 (93–95)	92 (89–95) 87 (86–88)	99 (97–100) 78 (74–83)
4	1+ Physician Visit CIRRHOSIS and 1+ Hospital Diagnosis	85 (78–92) 95 (95–96)	90 (87–93) 88 (87–89)	99 (97–100) 79 (75–83)
4b	1+ Physician Visit CIRRHOSIS and 1+ Hospital Diagnosis *(excluding K766 portal hypertension)*	79 (71–87) 98 (98–99)	79 (74–83) 95 (94–96)	89 (83–96) 89 (86–93)
5	1+ Physician Visit CIRRHOSIS and (1+ Hospital Diagnosis or 1+ Procedure)	86 (79–93) 95 (95–96)	91 (88–94) 88 (86–89)	99 (97–100) 79 (74–83)
6	1+ Physician Visit CIRRHOSIS and (1+ Hospital Diagnosis or 1+ Death Code or 1+ Procedure)	88 (81–94) 95 (94–96)	92 (89–95) 88 (86–89)	99 (97–100) 79 (74–83)

### Algorithms for hepatocellular carcinoma

The most sensitive algorithm for HCC was any of: two or more physician visit codes, a hospital diagnostic code, a death code or a procedure code with a physician visit code for cirrhosis (algorthim 9: sensitivity 96% in HBV, 97% in HCV, 96% in KHSC group, Table 5). The most specific algorithm not employing pathological data was one physician visit cirrhosis code and one procedure code (specificity 100% in both HBV and HCV, 97% in KHSC group). This compared favourably to the specificity of using the Ontario Cancer Registry (which includes pathology data), which had a specificity of 99% in HBV and HCV groups, 97% in KHSC group. The algorithm with the highest overall accuracy in the HBV (99%, CI 98–99), HCV (98%, CI 98–99%) and all-cause liver disease (96%, CI 94–98%) groups was a diagnosis in the Ontario Cancer Registry or a hospital diagnostic or death code for HCC.

**Table 5:** Administrative data algorithms used to identify patients with hepatocellular carcinoma. Number of patients in each group with hepatocellular carcinoma (reference outcome) indicated (n) at top of column. HBV = Hepatitis B Virus infection, HCV = Hepatitis C virus infection, TCLD = Toronto Centre for Liver Disease, KHSC = Kingston Health Sciences Centre, CLD = Chronic Liver Disease, Sens = sensitivity, Spec = specificity, CI = confidence interval. 1+ = code occurs on at least one date; 2+ = code occurs on at least two separate dates.

Algorithm		HBV patients, TCLD (n = 133)	HCV patients, TCLD (n = 167)	KHSC patients (n = 25)
		Sens (95%CI), % Spec (95%CI), %	Sens (95%CI), % Spec (95%CI), %	Sens (95%CI), % Spec (95%CI), %
1	1+ Physician Visit	83 (76–89) 95 (94–96)	84 (79–90) 85 (84–86)	96 (88–100) 91 (89–94)
2	2+ Physician Visit	74 (66–81) 99 (99–99)	75 (69–82) 96 (96–97)	88 (75–100) 95 (93–97)
3	1+ Hospital Diagnosis or 1+ Death Code	80 (62–78) 100 (99–100)	78 (72–85) 99 (99–99)	92 (81–100) 96 (94–98)
4	Diagnosis in Ontario Cancer Registry	81 (75–88) 99 (99–100)	82 (76–88) 99 (99–99)	76 (59–93) 97 (95–98)
5	Diagnosis in Ontario Cancer Registry or 1+ Hospital Diagnosis or 1+ Death Code	87 (82–93) 99 (99–100)	92 (87–96) 99 (98–99)	92 (81–100) 96 (94–98)
6	1 Physician Visit CIRRHOSIS and 1+ Procedure	46 (37–54) 100 (99–100)	58 (51–66) 100 (99–100)	40 (21–59) 97 (96–99)
7	1+ Hospital Diagnosis or 1+ death code or 1+ Physician Visit	88 (82–94) 95 (94–96)	90 (85–94) 85 (83–86)	100 (100–100) 91 (89–94)
8	1+ Hospital Diagnosis or 1+ Death Code or 2+ Physician Visits	82 (75–89) 99 (98–99)	86 (81–92) 96 (95–97)	96 (88–100) 94 (92–97)
9	1+ Hospital Diagnosis or 1+ Death Code or 2+ Physician Visits or (1+ Physician Visit CIRRHOSIS and 1+ Procedure)	96 (92–99) 98 (98–99)	97 (94–100) 96 (95–96)	96 (88–100) 93 (91–96)
10	1+ Hospital Diagnosis or 1+ Death Code or 2+ Physician Visit or 1+ Physician Visit CIRRHOSIS and 1+ Procedure) or Diagnosis in Ontario Cancer Registry	97 (94–1.00) 98 (98–0.99)	97 (94–100) 96 (95–96)	96 (88–100) 93 (91–96)

## Discussion

We identified sensitive and specific algorithms for the identification of cirrhosis, decompensated cirrhosis and HCC in patients with HBV or HCV infection, and confirmed these findings in patients with known liver disease of other causes. While identifying an algorithm that combines optimal sensitivity and specificity is desired, this is not always possible, and thus different criteria can be used depending on the study purpose. If the goal is to identify a cohort of individuals with a condition, highly specific criteria are preferable as this maximizes the likelihood that included individuals indeed have the target condition. In contrast, when the condition is the outcome variable in a study, a more sensitive definition can be used to prevent underestimation.

In our study, the tradeoff between sensitivity and specificity was most notable for cirrhosis. In particular, outpatient physician claims for cirrhosis were very sensitive but not specific. We believe this is because, until recently, physicians in Ontario could receive additional payment for a visit with a diagnosis of cirrhosis.[27, 28] On this basis, physicians may have been more likely to correctly list this diagnosis on their billing claim, or even to “up-code” patients with borderline clinical features consistent with cirrhosis. Although algorithms for cirrhosis and decompensated cirrhosis demonstrated inverse relationships between sensitivity and specificity, we were able to identify an algorithm for HCC that was both highly sensitive and specific.

Hospitalization diagnostic data are entered by trained chart abstractors and may be more reliable than outpatient physician billing claims. Hospitalization codes were more specific than physician visit codes for the target conditions in our study. The only exception was the in-hospital diagnostic code for portal hypertension, which falsely identified several patients as having decompensated cirrhosis. Non-cirrhotic portal hypertension is covered by this diagnostic code, as is portal hypertensive gastropathy, a condition diagnosed based on non-specific endoscopic findings, raising the possibility that it might be over-diagnosed. These conditions may explain this diagnostic code’s lack of specificity.

Overall, algorithms to identify complications in HBV-infected patients tended to be less sensitive but more specific than the same algorithms, when used in the HCV or KHSC groups. The difference was largest for algorithms identifying decompensated cirrhosis. We suspect that this can be explained by lower rates of hospital-based care for HBV-infected patients, who had fewer previous hospitalizations and emergency department visits than the other two groups. Substance use and mental health issues may contribute to greater healthcare usage by patients with HCV infection.[29] The range in results obtained in our study underscores the importance of testing administrative algorithms in several different patient populations. Although sensitivity, specificity and accuracy varied across patient groups, the most sensitive, specific and accurate algorithms in each group remained the same or very similar.

Previous studies have validated administrative data algorithms for cirrhosis, decompensation and HCC in U.S. populations. As we have done, others have also included codes for decompensation events as part of their administrative data definition of cirrhosis, or have combined HCC codes with cirrhosis codes to improve algorithm performance.[11, 12] In one validation study of cirrhosis definitions, sensitivity using multiple codes was achieved at the expense of low specificity, similar to what we observed.[15] While prior studies were limited to ICD-9 codes, ours is the first study to test the performance of ICD-10 codes for the detection of liver disease outcomes. U.S. hospital data have been coded using ICD-10 since 2015.[19] The algorithms and codes we provide can now be used to identify liver disease outcomes using Canadian, U.S. and European health administrative data, as well as any data coded in the widely-used ICD-9 or ICD-10 systems.

Strengths of our study are the inclusion of inpatient and outpatient data, as well as procedures, cause of death and pathology results, all tested against a physician-confirmed reference standard. Further, we included a large number of individuals in three patient cohorts from two institutions, with different etiologies of liver disease. The consistency of the relative ranking of algorithms across patient groups suggests that the most sensitive or specific algorithms can be applied broadly to all patients with known liver disease. Finally, our study results are comprehensive as they can be used to identify all three important clinical outcomes in patients with chronic liver disease.

Our study has several limitations. First, the existence of a premium payment for physician visits in Ontario may indicate that cirrhosis billing codes were claimed more often in our study setting than they would be elsewhere. Without a premium code, one might expect that a single physician visit code for cirrhosis would have greater specificity and lower sensitivity than measured in our study. Second, the study of hepatology clinic patients has advantages and disadvantages. One advantage is that specialist physicians with expertise in evaluating patients for liver outcomes can be expected to have greater diagnostic accuracy than generalist physicians. However, patients seen in a specialist clinic are likely to have more severe disease, which can lead to spectrum bias.[30] We would expect this to be most relevant for cirrhosis, where there is a clear spectrum of disease ranging from asymptomatic to severely symptomatic, decompensated cirrhosis.

If patients in a hepatology clinic are sicker than the general population, we would expect our measured sensitivity to be higher, and specificity to be lower, than they would be in the general population. Our results are valid for patients like those seen in a hepatology clinic: that is, patients with diagnosed liver diseases. Therefore, studies validating health administrative data codes against patient data abstracted from community hospitals, primary care clinics, or the general population would be an important contribution to the literature. In the general population, our specific algorithms can be used to define cohorts, however they may not be sensitive enough to identify all cases, and as such may underestimate outcome rates. An additional caveat is that some decompensation codes (e.g. ascites) can occur without liver disease. In order to avoid misclassification when applied to the general population, these codes should be combined with liver disease or cirrhosis codes.

## Conclusions

Liver diseases such as chronic HBV and HCV infection are a major cause of mortality worldwide. Administrative data can be used to identify large groups of patients with complications of chronic liver disease. We have reported the operating characteristics of algorithms for cirrhosis, decompensated cirrhosis, and hepatocellular carcinoma, using ICD-9 and ICD-10 health administrative data. We have identified sensitive and specific algorithms for each of these clinical conditions, which can be used to define patient cohorts or detect clinical outcomes. Our results will facilitate research into the adequacy of screening and treatment outcomes for patients with chronic HBV, HCV, or other liver diseases. Future research should test the performance of health administrative data codes in the general population.
